# Apparent Motion Perception in the Praying Mantis: Psychophysics and Modelling

**DOI:** 10.3390/vision2030032

**Published:** 2018-08-10

**Authors:** Ghaith Tarawneh, Lisa Jones, Vivek Nityananda, Ronny Rosner, Claire Rind, Jenny C. A. Read

**Affiliations:** Institute of Neuroscience, Newcastle University, Newcastle upon Tyne NE2 4HH, UK

**Keywords:** apparent motion, insect vision, Hassenstein-Reichardt detector, Dmax

## Abstract

**Simple Summary:**

Computer monitors, smart phone screens, and other forms of digital displays present a series of still images (frames) in which objects are displaced in small steps, tricking us into perceiving smooth motion. This illusion is referred to as “apparent motion”. For motion to be perceived, the magnitude of each displacement step must be smaller than a certain limit, referred to as Dmax. Previous studies have investigated the relationship between this limit and object size in humans and found that the maximum displacement is larger for larger objects than for smaller ones. In this work, we investigated the same relationship in the praying mantis *Sphodromantis lineola* by presenting them with moving random chequerboard patterns on a computer monitor. Even though motion perception in humans and insects are believed to be explained equally well by the same underlying model, we found that Dmax scales differently with object size in mantids. These results suggest that there may be qualitative differences in how mantids perceive apparent motion compared to humans.

**Abstract:**

Apparent motion is the perception of motion created by rapidly presenting still frames in which objects are displaced in space. Observers can reliably discriminate the direction of apparent motion when inter-frame object displacement is below a certain limit, Dmax. Earlier studies of motion perception in humans found that Dmax is lower-bounded at around 15 arcmin, and thereafter scales with the size of the spatial elements in the images. Here, we run corresponding experiments in the praying mantis *Sphodromantis lineola* to investigate how Dmax scales with the element size. We use random moving chequerboard patterns of varying element and displacement step sizes to elicit the optomotor response, a postural stabilization mechanism that causes mantids to lean in the direction of large-field motion. Subsequently, we calculate Dmax as the displacement step size corresponding to a 50% probability of detecting an optomotor response in the same direction as the stimulus. Our main findings are that the mantis Dmax scales roughly as a square-root of element size and that, in contrast to humans, it is not lower-bounded. We present two models to explain these observations: a simple high-level model based on motion energy in the Fourier domain and a more-detailed one based on the Reichardt Detector. The models present complementary intuitive and physiologically-realistic accounts of how Dmax scales with the element size in insects. We conclude that insect motion perception is limited by only a single stage of spatial filtering, reflecting the optics of the compound eye. In contrast, human motion perception reflects a second stage of spatial filtering, at coarser scales than imposed by human optics, likely corresponding to the magnocellular pathway. After this spatial filtering, mantis and human motion perception and Dmax are qualitatively very similar.

## 1. Introduction

The detection of motion is an important visual function in all animals. Movement can signal the presence of prey, a predator, or a conspecific in the animal’s surrounding environment and is therefore an important cue for many forms of behavior. To understand how motion detection in animal visual systems works, it is helpful to first examine and understand their responses to basic forms of visual input such as apparent motion stimuli. Apparent motion is an illusion of motion created by the rapid presentation of successive still frames in which objects are gradually displaced in space. The ability of an observer to perceive motion in such stimuli depends on a number of factors including the frame rate of presentation and the amount of spatial displacement between individual frames. When inter-frame displacement is made large, for example, objects no longer appear to be smoothly moving and are instead perceived to be jumping in discrete steps. Increasing inter-frame displacement further, beyond a certain limit, makes observers unable to determine the direction of motion. This limit is known as Dmax [1] and is a characteristic feature of a visual motion detection system. Measuring Dmax and examining its relationship with other stimuli features can reveal details about the computational mechanisms of motion detection in the visual system. It can also improve our understanding of how apparent motion is perceived and offer ways to improve video display technologies such as TVs and computer monitors that rely on this phenomenon.

In an early study of apparent motion perception in humans, subjects were presented with random dot patterns and their ability to identify a correlated region of dots that was uniformly displaced on each frame was tested [1]. The individual stimulus frames contained no cues about the region’s location, and so subjects could only distinguish it based on the motion of its constituent dots. The study found that the subjects could not detect motion when the region was displaced by more than 15 arcmins between frames, and that varying dot size in the range 2 to 11 arcmins had no effect on this result. The author postulated that humans have a constant Dmax of 15 arcmins that is imposed by the dimensions of elementary motion detectors in the human visual system. Subsequent studies, however, have shown that, while Dmax appears to be constant for element sizes of up to 15 arcmins (consistent with [1]), increasing the element size further caused a proportional increase in Dmax [2,3]. Absolute and relative Dmax values were presumed to be in favor of different explanations of how humans perceive motion [1,4,5] but were later shown to be accounted for by a simple model consisting of an initial spatial filter stage followed by a motion detection mechanism that is dependent on element size [3]. Although many studies have investigated Dmax and its implications in humans over the course of four decades [1,3,6], no corresponding studies have been reported in insects. Measuring the properties of Dmax would not only give an insight into the operation of visual motion detection in insects but would also enable comparison with humans, potentially shedding light on the evolutionary origins of motion perception.

In this study, we tested the apparent motion perception in the praying mantis *Sphodromantis lineola* using moving random chequerboard patterns rendered on a computer monitor. We measured Dmax for a range of element sizes and found that insect Dmax scales with element size. This scaling was observed in humans for large sizes, but whereas the human Dmax asymptoted to a fixed minimum at fine scales, the insect Dmax continued to decrease as the element size decreased, down to the smallest scales detectable by insects.

We present two computational models for the relationship between Dmax and element size in the mantis: (1) a simplified energy-based model [7] that assumes uniform spatiotemporal sensitivity but still accounts for the qualitative features of our results; and (2) a more-detailed model based on the Hassenstein Reichardt correlator or “Reichardt Detector” (RD) that is widely used to account for large field motion perception in insects [8,9] and is in good agreement with our observations, both qualitatively and quantitatively. Taken together, the models offer complementary insights into insect Dmax. The first offers an intuitive explanation for Dmax scaling based on motion energy distribution in the Fourier domain and the second demonstrates consistency with standard motion detection models.

## 2. Results

### 2.1. Experimental Findings

**Figure 1:**
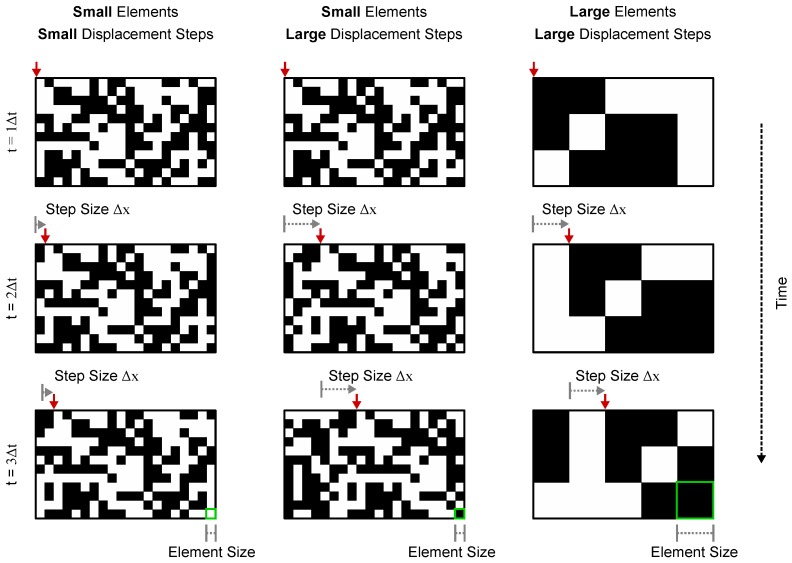
Examples of the random chequerboard pattern stimuli used in the experiment. Each column of panels shows still frames of a random chequerboard pattern that moves across a screen. The pattern is displaced by a fixed step (Δx) on Δt intervals where the speed v=Δx/Δt is constant (the red arrow at the top of each panel points to a reference point for easier visualization). The perception of motion in these patterns is strongly dependent on the displacement step (Δx) relative to the pattern’s chequer/element size. When Δx is comparable to the element size, humans perceive the pattern as moving smoothly and can identify its direction reliably (**column 1**). Increasing Δx causes the pattern motion to appear more “jerky” and makes its direction harder to identify (**column 2**). Because the perception of motion is dependent on the ratio between the element and step size, increasing the element size to match the step size makes the patterns appear to be moving smoothly again (**column 3**).

**Figure 2:**
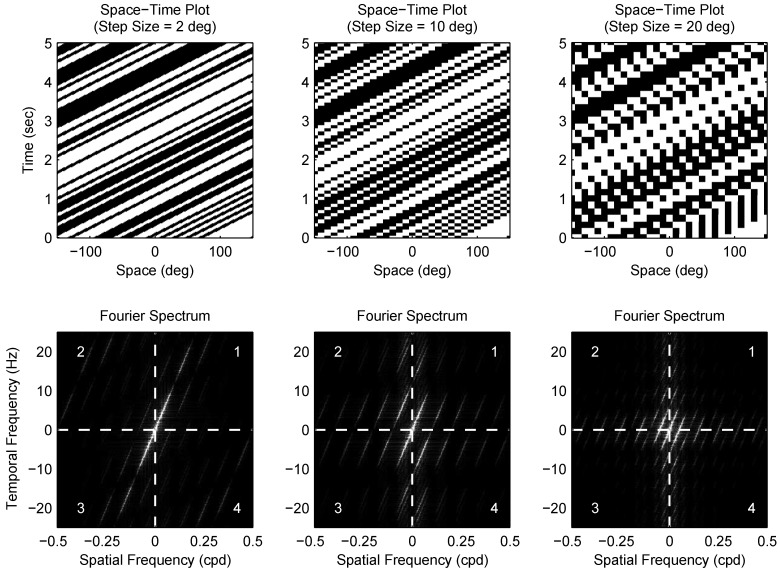
Spacetime plots and Fourier spectra for stimuli with different step sizes (10 deg elements). The top panels show space–time plots and the bottom panels show corresponding spatiotemporal Fourier spectra for a number of step sizes (the element size is 10 deg in all plots). When the step size is much less than the element size (left column), the stimulus is perceived by humans as moving smoothly and its motion energy is situated in quadrants 1 and 3 in the Fourier spectra (i.e., motion in the rightwards direction). In the middle column (step size equals element size), humans see the stimulus as moving less smoothly and part of its motion energy is now distributed in quadrants 2 and 4 in the Fourier domain (leftward motion). Finally, when the step size is much larger than the element size (right column), motion energy is more evenly distributed across the four Fourier quadrants, and humans find it significantly more difficult to identify the motion direction.

Mantids were placed in front of a Cathode Ray Tube (CRT) monitor and viewed a full screen random chequerboard stimulus moving horizontally in a series of steps. The size of the individual chequers (the *element size*) and the displacement magnitude in each step (the *step size*) were varied in different trials (Figure 1). When the step size was small, the stimulus appeared to humans as a smoothly moving pattern and elicited a postural stabilization mechanism (the optomotor response) in the mantis, causing them to lean in the direction of motion. As the step size was made larger, humans perceived the motion as jerky instead of smooth, and then at still larger step sizes, the motion percept was lost altogether, and the stimulus appeared as a sequence of random chequerboards with no relationship between them. In mantids, as the step size increased, stimuli ceased to elicit the optomotor response and mantids responded by either peering (moving the head and body from side to side, simultaneously counter-rotating the head so as to keep the eyes looking straight) or remaining still. In all trials, the time between steps was proportional to the step size, so the apparent motion speed was kept constant at 12.5 cm/s. Figure 2 shows stimulus space–time plots and corresponding spatiotemporal Fourier spectra for different step sizes.

An observer viewed the mantis through a web camera (while being blind to the stimulus) and coded its response in each trial as “moved left”, “moved right” or “other” (peering in both directions equally or no response) ([10]). The motion probability was subsequently calculated as the proportion of trials in which motion was detected (mantis leaned in the same direction as the stimulus) under each condition. This is the closest feasible analogy to human psychophysics experiments, where humans classify their own perceptual experience into discrete classes. We have shown previously that human observers very rarely code the mantis as moving in the opposite direction to the stimulus [10,11,12]. Consistent with our earlier work, we found that observers reported mantids to be moving in the opposite direction to the stimulus in only 4% of trials (this proportion was similar across all conditions).

**Figure 3:**
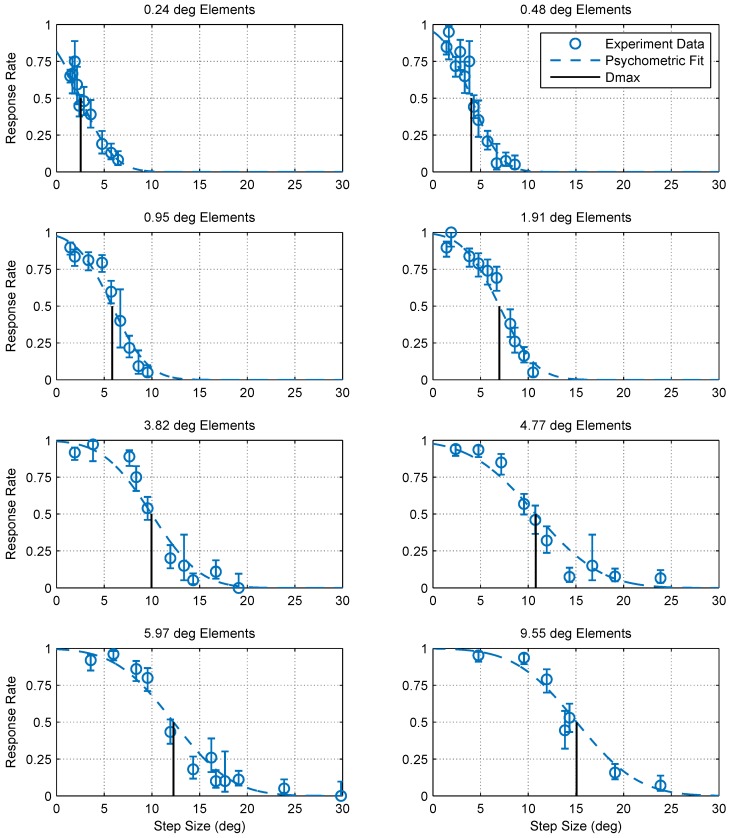
Optomotor response rates against step size for various element sizes. Error bars are 95% confidence intervals calculated using simple binomial statistics. Each panel shows the pooled responses of 13 mantids to a moving chequerboard stimulus of a given element size. The data and corresponding psychometric fits show that motion detection becomes increasingly more difficult (i.e., the response rate decreases) as the step size increases and the step size corresponding to a 50% response rate (Dmax) increases with element size.

**Figure 4:**
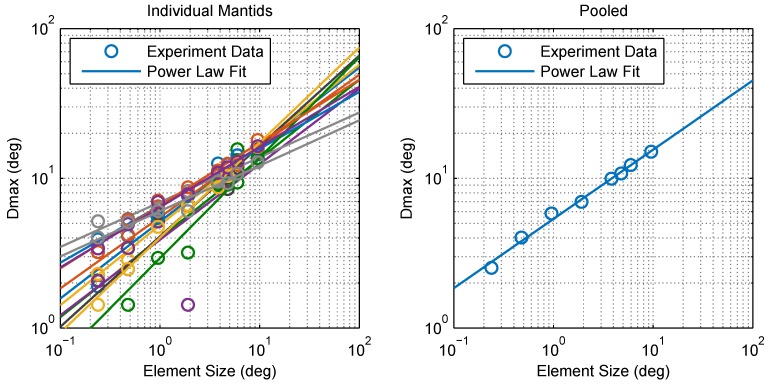
Relationship between Dmax and element size. Calculated Dmax values and power law fits for individual mantids (**left**) and pooled data (**right**). The points appear to lie on a straight line on this log-log plot indicating that Dmax can be described accurately as a power law function of element size *x*, determined via fitting as $\mathrm{Dmax}\left(x\right)=5.36\times x^{0.462}$ (standard error of the regression S=0.318${}^{\circ }$) for pooled data. Individual power law fits (**left**) had a mean *S* of 1.12${}^{\circ }$ with σ= 0.714${}^{\circ }$.

Figure 3 shows the collected data and fitted psychometric curves of motion probability versus the step size for different element sizes. From comparing the different panels in Figure 3, it is clear that increasing the element size shifts the psychometric curve in the positive direction of the step size axis. To quantify this change, we defined Dmax for each element size as the step size corresponding to a 50% probability of detecting motion, as estimated by a cumulative Gaussian psychometric function fit. Fifty percent corresponds to a 75% threshold on a two-alternative forced choice task where performance is at chance (50%) in the absence of a signal, and thus aligns with the human Dmax literature. At this point, thresholds are most accurate and least influenced by the shape of the psychometric function [13]. Figure 4 shows pooled and individual plots of Dmax versus the element size. We found that the relationship between Dmax and the element size is in good agreement (standard error of the regression *S* = 0.318${}^{\circ }$, over a range of 2–15${}^{\circ }$ in Dmax) with the following power law (whose parameters were determined using maximum likelihood fitting):

(1) $$\mathrm{Dmax}=5.36\times x^{0.462},$$

where *x* is element size in degrees.

### 2.2. Modeling and Simulation

#### 2.2.1. Model 1: Fourier Energy

Modifying a pattern moving smoothly at a given speed (*v*) by introducing a fixed step size (Δx) is equivalent to passing it through a “sample and hold” circuit with a sampling interval of Δt=Δx/v. The transformation introduces an aliasing artifact whereby temporal frequencies higher than 1/2Δt are cast into lower frequencies, as illustrated in Figure 2. A chequerboard pattern moving smoothly at speed *v* consist of a series of components, each with a spatial frequency ($f_{s}$) and a temporal frequency ($f_{t}$) where $f_{t}/f_{s}=v$ that lie in quadrants 1 and 3 of the spatiotemporal Fourier domain (for motion in the positive direction). When the pattern is moved in discrete steps, a fraction of its component energy is transferred to quadrants 2 and 4 (i.e., motion in the negative direction). Increasing the step size Δx (and correspondingly Δt, since speed is constant) causes a larger portion of pattern motion energy to be distributed across the four quadrants, making it more difficult to identify the direction of motion. This aliasing effect can be observed in the space–time plots of moving patterns. When motion is smooth, the space–time plot shows clear rightward-pointing structures, as in the left column of Figure 2. If the pattern is moved by a step size that is larger than the element size (right column), leftward-pointing structures emerge as a result of the false matches between non-corresponding blocks across different frames.

**Figure 5:**
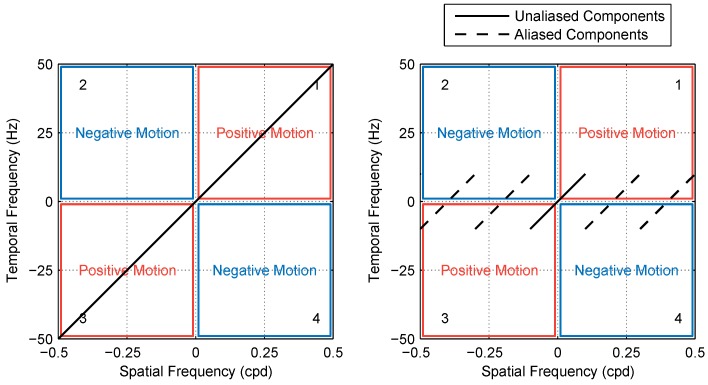
Effect of aliasing on the distribution of motion energy in the Fourier domain. A pattern moving coherently at a constant speed (*v*) has spatial and temporal frequency components along the line $f_{t}/f_{s}=v$, and its motion energy content is in quadrants 1 and 3 that signify positive motion (**left** panel). When the pattern is moved in steps of Δx at the same speed, components with temporal frequencies higher than v/Δx get aliased and cast towards lower frequencies (**right** panel). Aliased components are distributed in the four quadrants, and their net motion energy is therefore close to zero, leaving the energy of unaliased components as a close approximation of what remains in quadrants 1 and 3.

**Figure 6:**
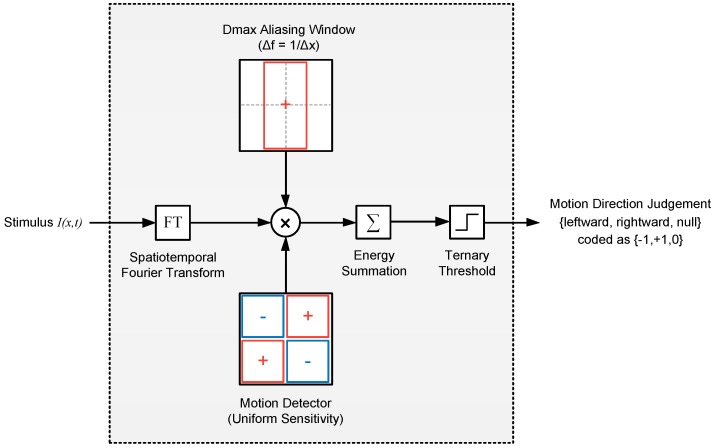
Apparent motion detection model based on Fourier Energy (Model 1). In this model, the spatiotemporal Fourier transform of the stimulus is filtered by a Dmax aliasing window (whose extent is Δf=1/Δx cpd, where Δx is presentation step size) and multiplied with the spatiotemporal sensitivity of an (ideal) motion detector. The net energy is then summed and passed through a ternary threshold to obtain a decision on the motion direction. The red and blue rectangles in filter blocks represent the spatiotemporal regions with positive and negative sensitivities, respectively.

**Figure 7:**
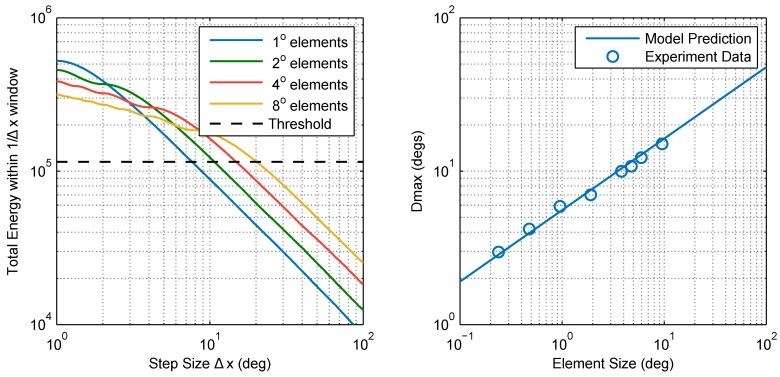
Simple motion energy-based model of Dmax in the praying mantis (Model 1). (**Left**) the total motion energy of unaliased pattern components contained within a frequency window ([−1/Δx,1/Δx]) decreases as the step size (Δx) is increased. Patterns of larger elements have a larger portion of their motion energy within lower frequencies and therefore contain more energy at large step sizes. According to this model, Dmax is the step size corresponding to a given motion energy threshold (chosen here to be ∼$10^{5}$ units); (**Right**) This simple model is in good agreement with our experimental results for the mantis (standard error of the regression, S=0.613${}^{\circ }$).

As a first order approximation, the motion energy of a stepped chequerboard pattern can be taken as the sum of its unaliased components. The rationale behind this is that components of any temporal frequency range [N−1/2,N+1/2]/Δt (where *N* is a non-zero natural number) are cast by aliasing to the range [−1/2,1/2]/Δt and so, assuming symmetry around N/Δt, would be split into two groups with equal energy in the positive and negative directions. The net motion energy of aliased components in the pattern can therefore be approximated as zero (Figure 5). We ran numerical simulations in which we calculated the motion energy of stimuli in our experiment using this model (shown as a block diagram in Figure 6). Figure 7 (left) shows a plot of approximated pattern energy versus step size Δx for element sizes in the range 1–8${}^{\circ }$. The plot shows that increasing element size causes a proportional shift in the curve over a region of energy levels (up to ∼$2\times 10^{5}$ units). Assuming the energy threshold (*T*) corresponding to Dmax is within this range (e.g., $T∼10^{5}$), we calculated Dmax as the step size at which motion energy crosses the threshold (for each element size). Figure 7 (right) shows a plot of the calculated Dmax versus the element size based on this model and compares this with our experimental findings. The predictions and experimental results are in good agreement, showing that this simple high-level model with a single parameter, the threshold (*T*), can account well for the Dmax values that we observed in the mantis.

#### 2.2.2. Model 2: Reichardt Detector

**Figure 8:**
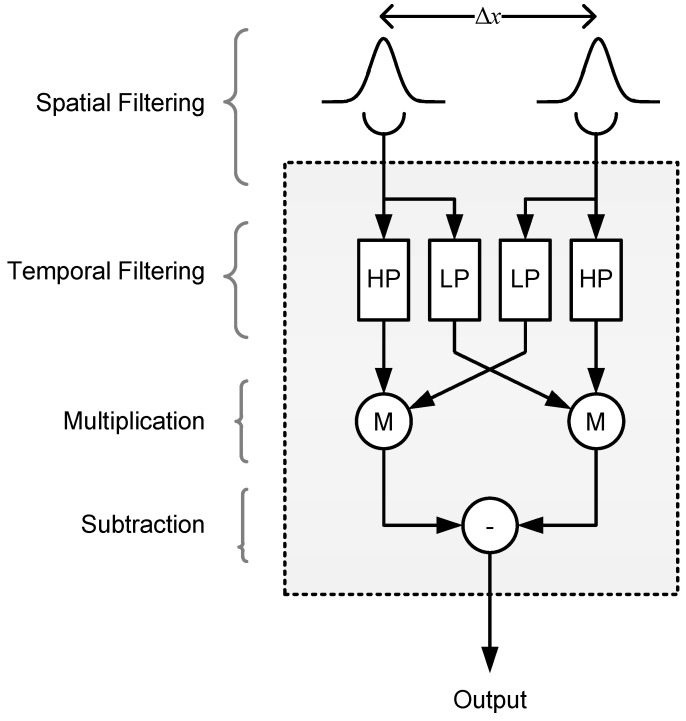
The Reichardt Detector. The spatial input from two identical Gaussian filters (standard deviation σ) separated by Δx is passed through high and low pass temporal filters (HP and LP, respectively). The LP output in each subunit is cross-correlated with the HP output from the other subunit using a multiplication stage (M), and the two products are then subtracted to produce a direction-sensitive measure of motion. In the RD-based Model 2 (Figure 9), the spatial filter parameters are $\sigma_{1}$ = 1.07${}^{\circ }$, $\Delta x_{1}$ = 1.0${}^{\circ }$ for detector class 1, and $\sigma_{2}$ = 3.42${}^{\circ }$, $\Delta x_{2}$ = 2.5${}^{\circ }$ for detector class 2, while the low and high pass filter time constants are $\tau_{L}$ = 13 ms, $\tau_{H}$ = 40 ms, respectively.

Model 1 is a simple and intuitive model which provides an excellent account of our data, but it does not explain how Fourier energy is computed in the mantis brain. More importantly, it fails to take account of the mantis spatiotemporal contrast sensitivity function [14], instead assuming that all Fourier components are equally visible. To verify that the intuitive account of Model 1 can survive a more realistic implementation, we built and simulated a more detailed, lower-level motion detection model to investigate whether our experimental findings could be explained by a system of narrow-band detectors. This second model is based on the well known Hassenstein Reichardt correlator or “Reichardt Detector” (RD) that is widely used to account for the optomotor response and similar large-field visually-guided forms of behavior in insects [8,15,16,17]. The detector (shown in Figure 8) consists of two subunits that compute motion in opposite directions, typically assumed to be neighboring ommatidia and their neural cartridges [18]. The spatial input from each subunit is temporally delayed and then compared with the other subunit’s input to detect luminance changes that have “travelled” in the same direction with a similar delay. The two subunit outputs are then summed to produce a direction-sensitive measure of motion.

Although the preferred spatial frequency of the detector can be varied by adjusting the spatial separation of its subunits and the extent of their input spatial filters, its spatial bandwidth is considerably constrained [11]. To enable the model to respond to stimuli covering a spatial bandwidth larger than that of the detector’s (such as the range of element sizes we tested), we combined multiple detectors tuned to two spatial frequencies (i.e., two detector *classes*). This is consistent with the existence of regional variations in ommatidia size and spacing across mantis eyes [19].

**Figure 9:**
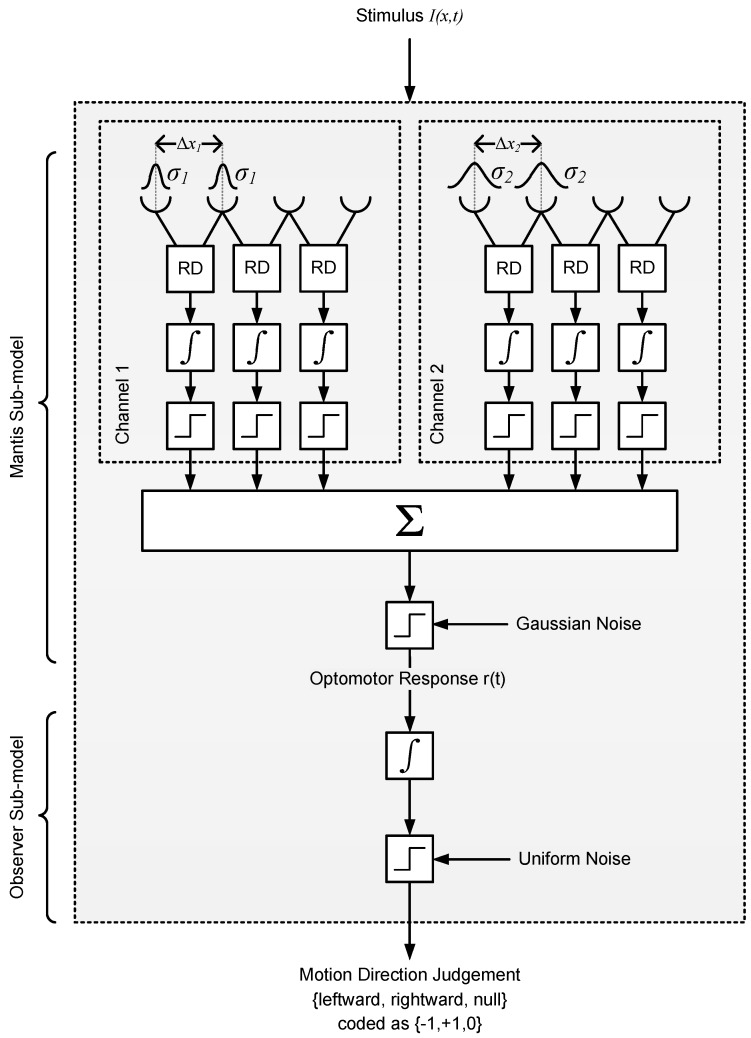
Optomotor response model based on the Reichardt Detector (Model 2). The model has two arrays of detectors that are positioned in random locations across a simulated 1D retina. Detectors within each array share the same subunit separation and spatial filter extent (and are thus all tuned to the same spatial frequency). Having two detector classes enables the model to respond to stimuli across a range of spatial frequencies broader than that of a single detector. The output of each detector is temporally integrated and then converted to a “vote” for leftward, rightward, or no motion by a hard threshold. The votes are subsequently combined, thresholded, and then passed through another temporal integrator and hard threshold blocks that model the decision process of the human observer in the experiment.

Our model is shown in Figure 9 and is essentially a slight variation of a published model of the mantis optomotor response [11]. The (free) parameters that we fitted were the separation and extent of spatial filters for each class ($\sigma_{1}$, $\sigma_{2}$, $\Delta x_{1}$, $\Delta x_{2}$), the two post-detector thresholds $T_{1}$ and $T_{2}$ (each for one of the two classes, normalized to maximum detector output), the mantis noise level (ξ) and the observer noise level (η) (both normalized to the maximum signal level).

The spatial parameters σ and Δx determine the spatial frequency tuning of each detector class (intuitively, larger Δx and σ values facilitate the detection of longer spatial periods and hence shift detector sensitivity towards lower spatial frequencies). Leaky integrator blocks calculate the average of each RD over the last 300 ms and pass this through hard two-sided thresholds to obtain “ternary votes” on the motion direction (−1, +1 or 0, corresponding to “moved left”, “moved right” or “other”). There are 25 detectors in each class (50 in total) and each class has its own threshold level ($T_{1}$, $T_{2}$). The votes are subsequently summed and passed through a noisy threshold block (using Gaussian noise) to calculate an optomotor response signal r(t) in the range [−1, 1]. Finally, r(t) is integrated over stimulus presentation and passed through a noisy ternary threshold to model the decision process of the human observer in the experiment.

The fitted parameter values were $\sigma_{1}$ = 1.07${}^{\circ }$, $\sigma_{2}$ = 3.42${}^{\circ }$, $\Delta x_{1}$ = 1.0${}^{\circ }$, $\Delta x_{2}$ = 2.5${}^{\circ }$, $T_{1}$ = 0.022, $T_{2}$ = 0.022, ξ = 0.0112 and η = 0.0588. Assuming the detector spatial filters correspond to individual ommatidia, the interommatidial angle (Δϕ=Δx) and acceptance angle (Δρ=2.35σ) of each class were $\Delta \phi_{1}$ = 1.0${}^{\circ }$, $\Delta \phi_{2}$ = 2.5${}^{\circ }$, $\Delta \rho_{1}$ = 2.5${}^{\circ }$, $\Delta \rho_{2}$ = 8${}^{\circ }$. These values are consistent with measurements of interommatidial parameters in the comparable mantis species *Tenodera australasiae* [19] and indicate that classes 1 and 2 are near-foveal and peripheral detectors, respectively. In addition to the fitted parameters, there were two other (non-free) parameters: the time constants of the RD’s low and high-pass temporal filters. Their values were selected as $\tau_{L}$ = 13 ms and $\tau_{H}$ = 40 ms based on physiologically plausible data reported in literature ([10,11,20]).

**Figure 10:**
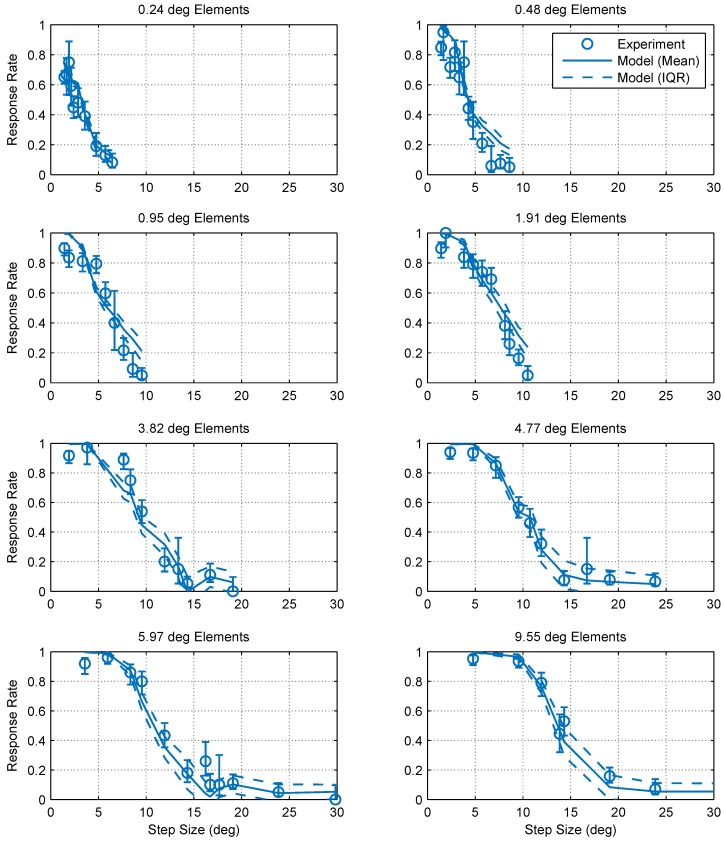
Predictions of Model 2 against experimental results. The mean (solid line) and interquartile range of Model 2 (dashed lines, based on 50 simulated trials per data point), showed good agreement with our experimental observations (symbols, with error-bars showing 95% confidence intervals).

Predictions were obtained by simulating the model using the same stimuli used in experiments. To reduce the simulation time, both RD spatial filters and stimuli had a single spatial dimension instead of two. Simulated trials lasted for 1 second, after which the model produced a ternary judgment: “moved left”, “moved right” or “other” (peering in both directions equally or no response), corresponding to the experiment. Figure 10 shows the predictions of this model versus the experiment data. The predictions are in good agreement with our experimental observations showing that, by combining at least two classes of detectors, mantis Dmax values can be explained by a system of narrow-band motion detection units.

#### 2.2.3. Model Comparison

**Figure 11:**
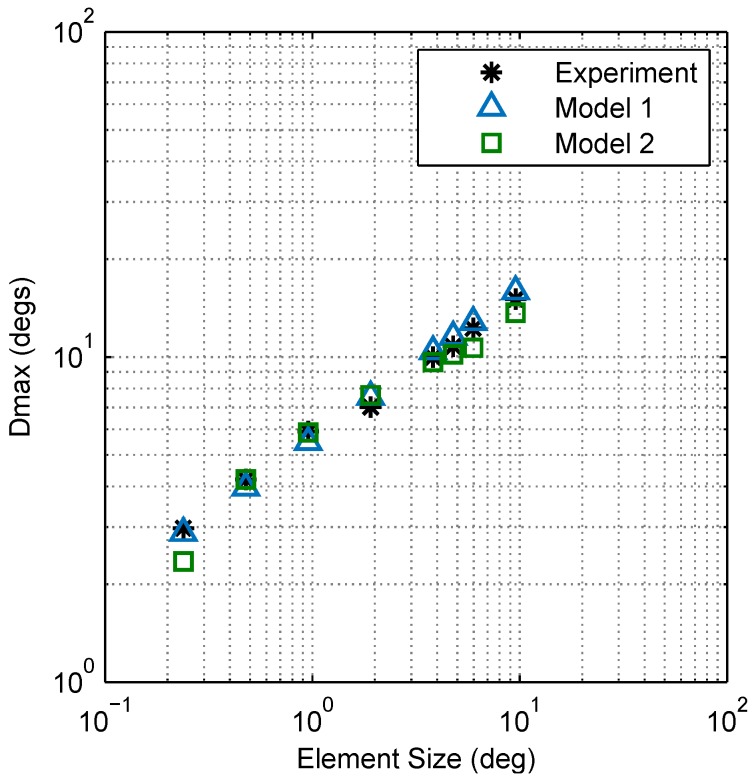
Comparison between Models 1 and 2. Plots of Dmax vs. element size for Models 1 and 2 show that both are in good agreement with experimental results (Model 1 has a standard error of the regression of S=0.613${}^{\circ }$ and Model 2 has S=0.967${}^{\circ }$).

Model 1, based on the Fourier spectra of stimuli, provides a good qualitative account of Dmax but assumes an underlying (ideal) motion detection system that is equally sensitive at all spatial and temporal frequencies. Published data of the mantis contrast sensitivity function, however, show maximum sensitivity in the spatial frequency range of 0.01 to 0.1 cycles per degree (cpd) and peak temporal sensitivity at 8 Hz [10]. In addition, there are significant regional variations in the size and spacing of ommatidia [19], the presumed structural correlates of motion detection spatial filters [9], making it likely that spatial sensitivity is also non-uniform across the visual field of the mantis. Since Model 1 did not take these details into account, it was important to cross-check the intuitive account of Dmax in terms of Fourier energy (derived from Model 1) against a more detailed model. In Model 2, we used a variation of the RD-based model from [11] to account for the spatial and temporal sensitivity of the mantis. We found it necessary to include two classes of RDs with distinct spatial tuning, since the bandwidth of any single detector class was too narrow to account for our experimental observations. Our results show that both Models 1 and 2 provide good accounts of experimental observations (Figure 11), consistent with the interpretation that (1) the mantis optomotor response depends on the total Fourier energy present within the stimulus; (2) mantis Dmax is imposed by aliasing artefacts which limit this energy and (3) spatiotemporal sensitivity does not significantly affect mantis Dmax provided that the stimulus is visible to the animal.

The Motion Energy model [7] is another prominent model that has been very successful in explaining a range of electro-physiological observations in mammals [21,22]. Even though the energy model relies on calculating Fourier energy, similar to Model 1, it includes spatial and temporal filters and can therefore account for spatiotemporal tuning, similar to Model 2 (it has in fact been proven to be formally equivalent to the RD under the assumption of separable filters [7]).

## 3. Discussion

Our results show similarities and differences between Dmax in humans and mantids. First, while the mantis Dmax appears to scale with element size similar to humans (for elements larger than 15 arcmin), we did not observe a region of element sizes with a constant Dmax (up to 15 arcmin in humans). Humans can perceive static dot patterns with much smaller element sizes, and so the lower bound on Dmax is evidence for the existence of a spatial filtration stage that precedes motion detection [3]. Consistent with this, applying spatial low pass filtration to random dot stimuli was found to increase the lowest Dmax bound [3]. Although it is possible that element sizes smaller than the ones tested may have a constant Dmax, the smallest element size in our experiment (0.24 deg) was smaller than the foveal interommatidial angle in the comparable mantis species *Tenodera australasiae* [19]. Mantids are also most sensitive to motion with spatial frequencies of 0.01∼0.1 cpd [10], a range much lower than the fundamental spatial frequency component of a chequerboard pattern with 1 deg elements (2 cpd). Patterns with elements as small as 0.24 deg can still be detected because of their lower frequency components, but as the element size decreases further, a larger portion of their energy shifts towards the highest spatial frequencies and outside the mantis sensitivity range. It is therefore likely that the smallest element size tested in this study was near the limit of mantis visual acuity. Consistent with this, our data (Figure 3) show that the response rate for 0.24 deg elements is significantly lower, even at the smallest step size, compared to larger elements. Mantids may, therefore, have no range of element sizes over which Dmax is constant. In other words, the highest spatial frequency experienced by the mantis visual system in these stimuli was already limited by their optics rather than by the element size. Thus, reducing the pattern size further would simply abolish the response rather than revealing a shift to constant Dmax. We conclude that mantids, unlike humans, do not show a range of element sizes over which Dmax is constant before it begins to rise.

**Figure 12:**
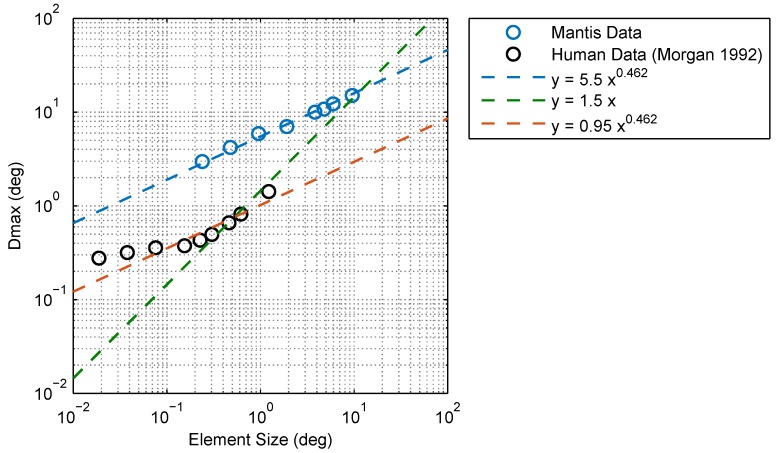
Comparison between Dmax in humans and mantids. Mantis Dmax increases with the element size, similar to humans, but does not appear to have a lower limit below which it remains constant (in humans, this limit is about 15 arcmin = 0.25 deg). The relationship between mantis Dmax and element size is well accounted for by a power law function with an exponent of 0.462 (blue line). Human data from [3] is plotted for comparison. Morgan interpreted the Dmax values he observed as scaling proportionately for element sizes larger than 0.25 deg (i.e., assumes a power law exponent of 1, green line) although his data appears to be accounted for equally well by a power law with an exponent of 0.462 (red line), similar to mantids. It is, therefore, unclear as to whether a true difference in power law exponents exists between humans and mantids.

Another apparent difference between mantis and human data concerns the relationship between Dmax and element size. In humans, Dmax is assumed to scale linearly with the element size (i.e., Dmax=kx for humans) [2,3,23,24] where the coefficient of proportionality (*k*) depends on a number of stimulus parameters, such as the spatial extent [5,25,26,27] and duration of presentation [26,28,29,30]. Our results in the mantis, however, appear to be best approximated by a power law fit with an exponent of of 0.462 (i.e., $\mathrm{Dmax}=kx^{0.462}$). Even though the coefficient of proportionality (*k*) is also likely to depend on the stimulus parameters, the different exponent may reflect a qualitative difference between motion processing in humans and mantids. However, a comparison between mantis and published human Dmax data does not make a compelling case for a qualitative difference (Figure 12). Fitting *k* to all the human data while constraining the exponent to be 0.462, as for mantids, produces a reasonable fit, even neglecting the lower bound on Dmax at small element sizes. In a more complex model accounting for this limit, it seems far from clear that an exponent of 1 would be the best fit. In fact, Dmax in both humans and insects may show a sublinear dependence on scale.

The ability of both our Fourier and RD-based models to reproduce the relationship between Dmax and element size in the mantis suggests further similarities. Our simple model based on stimulus energy in the Fourier domain is similar to the Motion Energy model used to account for apparent motion perception in humans [7]. Both assume that motion perception is computed by a cross-correlation operation in the space–time domain (or, equivalently, a summation of stimulus energy in the spatio-temporal Fourier domain). A direct implication of our model is that Dmax corresponds to a motion energy detection threshold that is reliably exceeded by smoothly moving stimuli but not by those with aliasing artifacts caused by large motion steps. According to this model, Dmax scales with the element size, because stimuli composed of larger elements have a greater portion of their energy within lower frequencies and can therefore be more severely aliased by larger step sizes before their unaliased component energy falls below the threshold. This simple account of Dmax is sufficient to explain the main characteristics of the relationship between Dmax and the element size but does not take into account the limited spatial sensitivity of local correlation-type detectors. The response properties predicted by these detectors were found to be in excellent agreement with a series of behavioral and physiological studies [31,32,33,34,35,36], and much is known about their neural substrates [20]. Reproducing the relationship between Dmax and the element size using the RD-based model was therefore important to verify that the intuition behind the Fourier model still holds when motion detection is mediated by units with narrow-band sensitivity and other physiologically-realistic details.

Because the spatial bandwidth of the Reichardt detector is considerably constrained, we found it necessary to use two detector classes with different levels of spatial tuning to replicate our experimental observations across the element size range tested (0.24–9.55${}^{\circ }$). Our stimulus subtended 142${}^{\circ }$ at the viewing distance of the mantis, an extent that spans significant variations in ommatidia size and spacing. It is therefore likely that the two detector classes in Model 2 correspond to different eye regions where ommatidia parameters (and hence detector spatial frequency tuning) are different. Reported measurements of ommatidial parameters in comparable mantis species *Tenodera australasiae* range from Δϕ = 0.6${}^{\circ }$, Δρ = 2${}^{\circ }$ in the fovea to Δϕ = 2.5${}^{\circ }$, Δρ = 6${}^{\circ }$ in peripheral eye regions, under dark-adapted conditions that match our experimental setup [19]. The fitted spatial filter parameters of Model 2 ($\Delta \phi_{1}$ = 1.0${}^{\circ }$, $\Delta \rho_{1}$ = 2.5${}^{\circ }$, $\Delta \phi_{2}$ = 2.5${}^{\circ }$, $\Delta \rho_{2}$ = 8${}^{\circ }$) are in reasonable agreement with these ranges and are consistent with the interpretation that classes 1 and 2 represent near-foveal and peripheral detectors, respectively. This interpretation makes the prediction that if stimulus presentation were limited to a subregion of the eye with little variation between ommatidia, then a model with a single detector class would explain the results. This prediction could be tested by, for example, painting over peripheral eye regions. Ref. [14] showed that the mantis optomotor response is driven predominantly by the central 50${}^{\circ }$ or so of the visual field, so even after this manipulation, the optomotor response should still be elicitable. This experiment would be a valuable test of our interpretation.

In conclusion, we observed that mantis Dmax scales sublinearly with the element size (in fact roughly as the square-root of element size, specifically $\mathrm{Dmax}=x^{0.462}$). Although human Dmax has been summarised as being proportional to element size, the human results were also consistent with a sublinear increase. We also found that two models based on the RD and motion energy (that are predominantly used in insect and human motion detection, respectively) provided consistent accounts of these experimental observations. The major qualitative difference between humans and insects is that human Dmax remains roughly constant for small element sizes before it begins its sublinear increase. We agree with [3] that this may reflect the distinct parvo- and magno-cellular pathways in human vision. In this account, human sensitivity to static patterns is mediated by the parvocellular pathway and has a high spatial-frequency limit matching the excellent optics of human eyes. Human motion perception, however, largely relies on magnocellular processing with a much lower spatial-frequency limit. Thus, in humans, Dmax initially reflects a coarse, magnocellular filter, even though humans can perceive much finer patterns using the parvocellular pathway. Only when the element size is coarser than the magnocellular filter does human Dmax begin to scale with element size. In insects, the spatial “sampling base” of the optomotor response fits the interommatidial angle [20] and so subsequent motion processsing stages, e.g., the pooling of Reichardt detectors into wide-field motion sensors apparently do not impose additional, coarser spatial filtering of the stimulus, at least not in the pathways mediating the optomotor response.

## 4. Methods

### 4.1. Insects

A total of 13 female mantids of the species *Sphodromantis lineola* were used in the experiment. Each individual was kept in a plastic box of dimensions 17×17×19 cm with a porous lid for ventilation and fed a live cricket twice per week. The boxes were kept at a temperature of 25${}^{\circ }$ and were cleaned and misted with water twice per week.

### 4.2. Experimental Setup

In each experiment, a mantis was hung upside down from a Perspex base and viewed full-screen stimuli on a CRT monitor (HP P1130). The base was held in place by a clamp such that the mantis was facing the middle point of the screen and the viewing distance (distance between mantis and monitor) was 7 cm in all trials. A web camera (Kinobo USB B3 HD Webcam) was placed under the base, providing a view of the mantis (but not the screen). The monitor, Perspex base and camera were all placed inside a wooden enclosure to isolate the mantis from distractions and to maintain consistent dark ambient lighting during experiments. The screen had physical dimensions of 40.4×30.2 cm and pixel dimensions of 1600×1200 pixels. At the viewing distance of the mantis, the horizontal extent of the monitor subtended a visual angle of 142${}^{\circ }$. The mean luminance of the monitor was 27.1 cd/m${}^{2}$, and its refresh rate was 85 Hz.

Experiments were programmed in Matlab 2012b (Mathworks, Inc., Natick, MA, USA) and stimuli developed using Psychophysics Toolbox Version 3 (PTB-3) [37,38,39] were used. The experiment computer was a Dell Optiplex 9010 (Dell Corporation Limited, Bracknell, UK) with an Nvidia Quadro K600 graphics card running Microsoft Windows 7.

### 4.3. Experimental Procedure

Each experiment consisted of a number of randomly-interleaved trials in which a mantis was presented with moving chequerboard patterns. An experimenter observed the mantis through the camera underneath and blindly coded the direction of the elicited optomotor response (if any). The response code for each trial was “moved left”, “moved right” or “other” (peering in both directions equally or no response). There were equal repeats of left-moving and right-moving patterns of each condition in all experiments. At the beginning of each trial, an alignment stimulus was used to steer the mantis back such that it was facing the middle point of the screen. The alignment stimulus was a smoothly moving chequerboard pattern controlled by the experimenter using a keyboard.

### 4.4. Visual Stimulus

The stimulus was a full-screen chequerboard pattern moving either leftwards or rightwards in each trial. The pattern was composed of black and white chequers of 0.161 and 54.1 cd/m${}^{2}$ luminance levels, respectively, chosen randomly with equal probability. The whole pattern was displaced horizontally by a step (Δx) at Δt intervals where Δx/Δt was kept constant across the different conditions. The stimulus speed was 12.5 cm/sec, corresponding to an optic flow of 44.4 deg/sec (averaged over screen width) at the viewing distance of the mantis (7 cm).

### 4.5. Calculating Dmax

We used likelihood fitting to calculate Dmax as the step size corresponding to a 50% response rate for each element size. The fitted psychometric functions were of the form

(2) $$P\left(\Delta x;\mathrm{Dmax},\sigma \right)=\frac{1}{2}\left(1-\mathrm{erf}\left(\frac{\Delta x-\mathrm{Dmax}}{\sqrt{2}\sigma }\right)\right),$$

where *p* is the probability that motion is detected, Δx is the step size, and σ is a parameter that specifies the width of the function’s transition period. The parameters Dmax and σ were calculated for each element size using maximum likelihood estimation in Matlab, assuming mantids’ responses had a simple binomial distribution. Therefore,

(3) (Dmax,σ)=argmin∑i=1klognimi+milogPi+ni−milog1−Pi,

where *k* is the number of step sizes, $n_{i}$ is the number of trials in which mantids detected motion, $m_{i}$ is the total number of trials, and $P_{i}$ is motion detection probability predicted by given Dmax and σ values, as per Figure 2. We calculated Dmax and σ for each insect’s individual data and for all insects pooled together.

### 4.6. Simulation and Model Fitting

#### 4.6.1. Model 1

Model 1 was simulated by calculating the Fourier transform of sample visual stimuli identical to those used in the experiment and summing their energy in the Fourier domain. This was done using the functions fft and trapz in Matlab (Mathworks Inc, Natick, MA, USA). The threshold level (*T*) was fitted in Matlab using the function fminsearch.

#### 4.6.2. Model 2

In RD simulations, we used Gaussian spatial filters and low/high pass temporal filters of the form

(4) $$\begin{array}{cc} TF_{1}\left(t\right) & =\exp(-t/\tau_{L}) \end{array}$$

(5) $$\begin{array}{cc} TF_{2}\left(t\right) & =\delta \left(t\right)-\exp\left(-t/\tau_{H}\right) \end{array}$$

(6) $$\begin{array}{cc} SF_{1}\left(x\right) & =\exp\left(\frac{-{\left(x-x_{0}-\Delta x/2\right)}^{2}}{2\sigma^{2}}\right) \end{array}$$

(7) $$\begin{array}{cc} SF_{2}\left(x\right) & =\exp\left(\frac{-{\left(x-x_{0}+\Delta x/2\right)}^{2}}{2\sigma^{2}}\right), \end{array}$$

where $\tau_{L}$ = 13 ms, $\tau_{H}$ = 40 ms, Δx, σ depended on the detector class ($\Delta x_{1}$ = 1.0${}^{\circ }$, $\Delta x_{2}$ = 2.5${}^{\circ }$, $\sigma_{1}$ = 1.07${}^{\circ }$, $\sigma_{2}$ = 3.42${}^{\circ }$), and $x_{0}$ was the detector position on the simulated retina. The models were normalized such that all gave a mean response of 1 to a drifting grating at the optimal spatial and temporal frequencies.

Model 2 was fitted using the optimization function fminsearch in Matlab (Mathworks, Inc., Natick, MA, USA). Repeated fitting attempts from randomly selecting initial solutions resulted in the same final solution (within numerical error) in the overwhelming majority of cases, confirming that the algorithm had located the global optimum rather than being stuck at a local minimum.

## 5. Data Availability

The data collected in this study are available on https://github.com/m3project/mantis-dmax.
